# When is it subfunctionalization and when is it not?

**DOI:** 10.1093/g3journal/jkae269

**Published:** 2024-12-19

**Authors:** James A Birchler

**Affiliations:** Division of Biological Sciences, University of Missouri, Columbia, MO 65211, USA

**Keywords:** gene duplication, subfunctionalization, neofunctionalization, whole genome duplication, small scale duplication

## Abstract

New genes arise from duplication followed by divergence. The most common fate of a duplication is one copy is deleted to return to a single copy. But if divergence occurs, the function of the gene can be split between the two copies, which is called subfunctionalization, or a new function can arise, which is called neofunctionalization. Changes in expression between the two copies does not necessarily suggest that subfunctionalization has occurred. In this commentary, the caveats and criteria for subfunctionalization are discussed.

Mutations generate new alleles and gene duplications are required to make new genes. How these two types of events interact can affect evolutionary processes with the role of gene duplication being long recognized (Ohno 1970; Lynch and Conery 2000). Gene duplications can arise from two paths that have different consequences (Hakes *et al*. 2007; Freeling 2009). One is Whole Genome Duplication (WGD) (or polyploidy), while the other is small scale duplications in tandem or transpositions from one genomic position to another. The most common fate of a duplication is that one or the other copy present is deleted wholly or partially resulting in a return to the singleton state (Freeling 2009). Following WGD, genes encoding members of molecular multisubunit complexes and highly connected in the interactome are retained for longer periods of evolutionary time than other classes of genes, presumably because there is a fitness cost of deletion of a single member of the complex rendering the whole complex less effective (Defoort *et al*. 2019). The complementary pattern is that these same classes of genes are underrepresented in small scale duplications, arguably because their unbalanced relationship with interactors has a fitness cost (Freeling 2009; Tasdighian *et al*. 2017). This relationship parallels experimental observations of “genetic balance” in which aneuploidy, or segmental changes in dosage, are more detrimental to the organism than changing the dosage of the whole genome via polyploidy and which is a reflection of stoichiometric changes in gene regulatory processes (Birchler and Veitia 2021). The fate of small scale cases can also be affected by whether the duplication event was RNA or DNA mediated (Hakes *et al*. 2007).

The duplications that survive deletion can diverge between the two copies and provide new functions to an organism (neofunctionalization) or the function of the progenitor can be divided (subfunctionalization) (Lynch and Conery 2000). This is grist for evolutionary novelty. However, there can be great variation in expression even for singleton genes, so deciding whether the division of a progenitor function (subfunctionalization) or the emergence of a new function (neofunctionalization) becomes a difficult determination to make.

Different alleles of a singleton gene can have different tissue specificity or quantitative expression, for example, that may be essentially neutral, or each specificity might have a selective advantage in a different environmental context. When examining the expression of a duplicate pair, different members might have a different tissue specificity or expression level and many authors consider this as evidence for subfunctionalization. It is possible however, that it is merely neutral variation in expression. A gene's function might be most critical in a particular cell type and less so or irrelevant in others. A duplicate pair can show compensatory drift in expression between the two members while maintaining the same total expression without proceeding to subfunctionalization (Thompson *et al*. 2016). Subfunctionalization in the strict sense is when the progenitor functions are divided without a path to return to the singleton state. Deletion of one or the other copy would have fitness cost with consequent fixation of the duplicate pair in the evolutionary lineage. Determining whether the variation of the members of a duplicate pair is merely neutral variation, compensatory drift, quantitative expression constraint, or an actual splitting of a function is devilishly difficult to determine at any one time point. Indeed, without sequence information of the two copies, it is entirely possible that a cryptic sub or neofunctionalization might have occurred even without a change of cell specificity or expression levels, for example, if the substrate specificity of an enzyme changes or alternative splicing might introduce changes without altering expression levels. One might posit that waiting 20 million years to see if the duplication is retained would suffice! Nevertheless, there are examples of subfunctionalization that can be identified. For example, fetal and adult hemoglobin are obviously descended from a common progenitor and now occupy different spaces in development.

Another complication in making this distinction is hypofunctionalization (Qian *et al*. 2010; Birchler and Veitia 2021). This is the situation when the collective expression from a duplicate pair mutates to a level that is subpar for the specified function. The members of the pair may be expressed at different levels but the total expression is less than the singleton progenitor. If experimental manipulation is possible, one might delete one member of the pair, find detrimental consequences, and come to the conclusion that subfunctionalization has occurred. In reality, the total expression drops further below that required for normal function of the cell without there being a splitting of functions. Hypofunctionalization might hold duplicates in an evolutionary lineage for an extended period, but further mutations can provide a return route to the singleton state (Qian *et al*. 2010) If members of a duplicate pair are expressed differentially *in different cell types* where there is no overlap and deletion of either one of them is detrimental, a conclusion of subfunctionalization is warranted.

It should be noted that the longer preservation of duplicate genes following WGD due to balance considerations will in fact hold the duplicate pair in the evolutionary lineage for longer periods of time (Birchler *et al*. 2005). Thus, retention of a duplicate pair per se is not necessarily due to a change of function. Yet, the balance constraint on deletion might allow an extended period for subsequent subfunctionalization or neofunctionalization to occur. Indeed, the classes of genes typically retained include transcription factors and signal transduction components, which are the ones that might have the most consequence from diverged or split functions. Because retained duplicate pairs decrease in number following WGDs going back in evolutionary time (Freeling 2009; Tasdighian *et al*. 2017), there is every reason to believe that the balance constraints will adjust over time and allow deletion back to the singleton state (Birchler *et al*. 2005; Birchler and Veitia 2021). Those pairs that continue to persist are excellent candidates for sub and neofunctionalization. A theoretical study concluded that dosage balance from WGD initially leads to a delay in subfunctionalization, but eventually fosters greater retention of duplicates, but for small scale duplicates, dosage balance reduces the chances for subfunctionalization (Wilson and Liberles 2023).

As noted, conclusions about neo or subfunctionalization occurring with a duplicate gene pair are not often as straightforward as many assume. Yet, clearly when they do occur, they generate evolutionary novelty. Changes in expression levels or tissue specificity alone do not necessarily signal sub or neofunctionalization, but they can also occur without either of these changes. Thus, caution should be exercised in concluding they occur.
